# Interfacility Variation in Diagnosis and Tocolysis for Threatened Preterm Labor and Its Association With Gestational Age Threshold for Neonatal Intensive Care Unit Admission: A Cross‐Sectional Survey of Perinatal Centers in the Chugoku–Shikoku Region of Western Japan

**DOI:** 10.1111/jog.70547

**Published:** 2026-10-28

**Authors:** Naofumi Yamane, Yuko Teraoka, Yurika Mukai, Kousuke Nakamoto, Yusuke Matoba, Yuriko Oomori, Katsuyuki Tomono, Tomomi Yamazaki, Kouji Banno, Ken Yamaguchi

**Affiliations:** ^1^ Department of Obstetrics and Gynecology Hiroshima University Hospital Hiroshima Japan; ^2^ Department of Obstetrics and Gynecology Miyoshi Central Hospital Hiroshima Japan; ^3^ Department of Obstetrics and Gynecology Hiroshima Prefectural Hospital Hiroshima Japan; ^4^ Department of Obstetrics and Gynecology Graduate School of Biomedical and Health Sciences Hiroshima Japan

**Keywords:** neonatal intensive care units, quality of health care, surveys and questionnaires, threatened preterm labor, tocolytic agents

## Abstract

**Aim:**

To describe interfacility variation in threatened preterm labor (TPL) management in a perinatal care network in western Japan and the association between the neonatal intensive care unit (NICU) admission threshold and the long‐term tocolysis policy.

**Methods:**

A cross‐sectional, facility‐level questionnaire survey was conducted for all 43 perinatal maternal and child health centers in the Chugoku–Shikoku region of western Japan in 2024. Centers reported diagnostic criteria for TPL, use of objective scoring systems, first‐line intravenous tocolytic drugs, NICU admission gestational age threshold, and institutional policies regarding planned duration of intravenous tocolysis (short‐term, ≤ 48 h; long‐term, > 48 h). Factors related to the adoption of long‐term tocolysis were evaluated using logistic regression.

**Results:**

Valid responses were obtained from 32 centers (74.4%). A long‐term‐only tocolysis policy was reported by 65.6% (21/32) of centers. Diagnostic criteria and thresholds for intervention varied widely, and only three centers (9.4%) used an objective severity score, such as the preterm labor index or tocolysis index. Ritodrine hydrochloride was used as the first‐line intravenous tocolytic drug in 93.8% (30/32) of centers. The NICU admission threshold differed among centers. In multivariable analysis, centers with a higher NICU admission threshold were more likely to report a long‐term‐only policy (adjusted odds ratio, 1.38 per 1‐week increase; 95% confidence interval, 1.04–1.83; *p* = 0.028).

**Conclusions:**

Management of TPL showed marked interfacility variation in the regional perinatal care network. The NICU admission threshold was associated with the institutional tocolysis‐duration policy, suggesting that regional perinatal system structure should be considered when discussing optimal tocolysis duration.

## 1 Introduction

Preterm birth is a major cause of neonatal mortality and long‐term morbidity. Although Japan has a relatively low overall preterm birth rate, extremely preterm birth remains a clinical challenge [1]. Management of threatened preterm labor (TPL) (regular uterine contractions with cervical change) often includes tocolysis, but international guidance generally positions tocolysis as a short‐term measure to allow administration of antenatal corticosteroids and/or maternal transfer, rather than as maintenance therapy aimed at prolonging gestation [2, 3]. Long‐term intravenous ritodrine hydrochloride infusion has historically been common in Japan, despite concerns about maternal adverse events and differences from international practice [4, 5, 6].

Because perinatal care is organized regionally, clinical decisions in obstetrics may be influenced by neonatal intensive care capacity and admission criteria, in addition to obstetric evidence [4]. However, there are limited facility‐level data showing how the gestational age threshold for neonatal intensive care unit (NICU) admission relates to the tocolysis strategy. Therefore, we conducted a questionnaire‐based regional facility survey of perinatal centers in the Chugoku–Shikoku region to examine diagnostic criteria and tocolysis use for TPL, and to determine whether the gestational age threshold for NICU admission is associated with use of long‐term intravenous tocolysis.

## 2 Materials and Methods

### 2.1 Study Design and Setting

A cross‐sectional, facility‐level questionnaire survey was conducted among all perinatal maternal and child health centers (comprehensive and regional) in the Chugoku–Shikoku region of Japan: 43 centers across Okayama, Hiroshima, Yamaguchi, Shimane, Tottori, Ehime, Tokushima, Kagawa, and Kochi prefectures. The survey period covered obstetric care delivered between January 1 and December 31, 2024. A paper invitation letter and a link to a web‐based questionnaire (Google Forms) were sent to the chief obstetrician at each center on February 10, 2025. Respondents were asked to complete the questionnaire online by February 28, 2025; nonresponders received one reminder. The questionnaire collected aggregated facility‐level data; no individual patient identifiers (names, hospital IDs, dates of birth, etc.) were collected. If duplicate responses were received, the most recent response was used and discrepancies were resolved by contacting the centers. Additional facility‐level information on formally registered NICU/GCU bed counts, antepartum transfer to lower‐level centers, and planned tocolysis for > 48 h as a bridge to transfer was obtained from all 32 participating centers.

### 2.2 Data Collected and Variable Definitions

A structured questionnaire was used to collect the following information:

*Facility characteristics*: Region (Chugoku vs. Shikoku), annual numbers of deliveries, numbers of deliveries < 34 and < 28 weeks, numbers of formally registered NICU and growing care unit (GCU) beds eligible for reimbursement, gestational age from which the NICU accepts admissions (“NICU admission gestational age threshold”), facility workforce (e.g., number of full‐time obstetricians and neonatologists), the requirement for maternal transfer to higher‐level centers, and the practice of antepartum transfer to lower‐level centers. Formally registered beds were distinguished from nonregistered beds providing functionally equivalent neonatal care, and planned tocolysis for > 48 h as a bridge to transfer to a lower‐level center was recorded as a facility policy.

*Diagnostic criteria for TPL*: Clinical findings used (uterine contractions, cervical dilation, cervical length, vaginal bleeding), cut‐off values for cervical length, and use of objective indices (preterm labor index [PLI] or tocolysis index [TI]).

*Tocolysis policy and drug use*: First and second choice tocolytic agents (generic names; e.g., ritodrine hydrochloride, magnesium sulfate, nifedipine), institutional policy for planned duration of intravenous tocolysis, and whether intravenous tocolysis is continued beyond 34 weeks of gestation. Based on international guidelines and clinical considerations, short‐term tocolysis was defined as administration of an intravenous tocolytic agent for ≤ 48 h to facilitate maternal transfer or completion of antenatal corticosteroid therapy, and long‐term (maintenance) tocolysis as use of an intravenous tocolytic agent for > 48 h with the aim of prolonging the pregnancy itself. Classification was based on each center's self‐reported policy for the planned duration of tocolysis at treatment initiation, rather than actual treatment duration or individual patient outcomes. For descriptive purposes, centers were categorized into three groups according to their routine intravenous tocolysis strategy: centers reporting a planned duration of ≤ 48 h only were classified as short‐term only; those reporting both ≤ 48 and > 48‐h strategies on a case‐by‐case basis as short‐term plus long‐term; and those reporting a planned duration of > 48 h only as long‐term only. For the primary analyses, centers were dichotomized into short‐term (short‐term only and short‐term plus long‐term) and long‐term (long‐term only) groups to assess whether short‐term tocolysis had been adopted as an institutional option.

### 2.3 Statistical Analysis

Continuous variables are reported as medians with interquartile ranges (IQR) and compared between groups by Mann–Whitney *U* test. Categorical variables are shown as numbers (percentages) and compared by *χ* ^2^ test or Fisher exact test, as appropriate. Facility‐level factors associated with use of long‐term tocolysis were identified by multivariable logistic regression. Candidate predictors were chosen a priori based on clinical relevance and to avoid overfitting given the sample size (*n* = 32): NICU admission gestational age threshold (modeled per 1‐week increase), number of formally registered NICU beds (modeled per 1‐bed increase) and region (Shikoku vs. Chugoku). Odds ratios (ORs) with 95% confidence intervals (CI) are reported, with two‐sided *p* values < 0.05 considered significant. An exploratory univariate analysis was conducted to compare the facility workforce and the requirement for maternal transfer between the short‐term and long‐term tocolysis groups. Sensitivity analyses grouped short‐term‐plus‐long‐term centers with long‐term‐only centers or excluded mixed‐policy centers. EZR v.1.61 (Saitama Medical Center, Jichi Medical University, Saitama, Japan), a graphical user interface for R7, was used for all analyses [7].

### 2.4 Ethics, Consent, Data Availability and AI Tools

This study used aggregated, facility‐level data and did not involve direct patient contact. The study protocol was approved by the Ethical Committee for Epidemiology of Hiroshima University (approval no. E2025‐0194). Completion of the questionnaire was taken to indicate consent to participate. Because combinations of the facility‐level variables could permit re‐identification of participating centers, the underlying dataset is not publicly available; the restrictions governing data access are described in the Data Availability Statement. No generative AI or large language model tools were used for data collection, analysis, or manuscript preparation.

## 3 Results

### 3.1 Facility Characteristics and Obstetric Volume

Responses were received from 32 of the 43 perinatal maternal and child health centers (response rate = 74.4%), including 10 comprehensive centers and 22 regional centers (Table S1 and Figure S1). For the primary analyses, centers were classified into a long‐term tocolysis group (long‐term only; 21/32, 65.6%) and a short‐term tocolysis group (11/32, 34.4%), comprising short‐term only (4/32, 12.5%) and short‐term plus long‐term (7/32, 21.9%) centers.

The 32 centers had 12 499 deliveries in 2024, including 469 (3.8%) preterm births at < 34 weeks, 66 (0.5%) extremely preterm births at < 28 weeks, and 877 cases (7.0% of all deliveries) diagnosed with TPL and managed with intravenous tocolysis. NICU admission thresholds were 22–23 weeks at 13/32 centers (40.6%), 24–27 weeks at 2/32 (6.3%), 28–33 weeks at 9/32 (28.1%), and ≥ 34 weeks at 8/32 (25.0%). Comprehensive centers accounted for 69.2% (9/13) and 50.0% (1/2) of centers accepting admissions at 22–23 and 24–27 weeks, respectively. All centers with admission thresholds of 28–33 weeks (9/9) or ≥ 34 weeks (8/8) were regional centers (Table S2). The median numbers of formally registered NICU and GCU beds were 6 (IQR, 5.3–12) and 6 (IQR, 0–12), respectively (Table 1).

**TABLE 1:** Characteristics of perinatal centers and comparison by routine intravenous tocolysis duration policy.

Characteristic	Total (*n* = 32)	Short‐term tocolysis (*n* = 11)	Long‐term tocolysis (*n* = 21)	*p*
Region, *n* (%)				0.70^a^
Chugoku	22 (68.8)	7 (63.6)	15 (71.4)	
Shikoku	10 (31.3)	4 (36.4)	6 (28.6)	
Annual deliveries, median (IQR)	428 (289–507)	456 (270–610)	382 (290–471)	0.50^b^
Preterm birth rate < 34 weeks (%), median (IQR)	2.5 (1.4–6.0)	7.3 (2.2–14.1)	2.1 (0.3–4.1)	**0.026** ^**b**^
Formally registered NICU beds, median (IQR)	6 (5.3–12)	9 (6–12)	6 (3–12)	0.320^b^
Formally registered GCU beds, median (IQR)	6 (0–12)	9 (3–16.5)	0 (0–10)	0.077^b^
Gestational age threshold for NICU admission (weeks), median (IQR)	28 (22–32)	22 (22–26)	30 (22–34)	**0.021** ^**b**^

*Note:* Bold values indicate statistical significance (*p* < 0.05).

Abbreviations: GCU, growing care unit; IQR, interquartile range; NICU, neonatal intensive care unit.

^a^ Fisher's exact test.

^b^ Mann–Whitney *U* test.

The workforce and requirement for maternal transfer in each facility were examined in an exploratory analysis (Table S3). Facilities with a short‐term tocolysis policy had significantly more full‐time obstetricians and neonatologists than those with a long‐term policy. The long‐term group had a higher proportion of centers requiring maternal transfer to higher‐level centers (76% vs. 45%), although the difference was not statistically significant. Among the 21 facilities that required maternal transfer, the median transfer distance to a higher‐level center did not differ significantly between the short‐term and long‐term groups (11.7 vs. 33.9 km, *p* = 0.905). Antepartum transfer to a lower‐level center was reported by 20/32 centers (9/10 comprehensive and 11/22 regional). Of the 20 centers that performed antepartum transfer to a lower‐level center, 14 had a policy of continuing intravenous tocolysis for > 48 h as a bridge to transfer, either routinely or depending on the case. These 14 centers comprised four comprehensive and 10 regional centers.

### 3.2 Heterogeneity of Diagnostic Criteria and Limited Use of Objective Indices

All 32 centers reported using cervical length measurement and cervical dilation as diagnostic indicators for TPL. Painful uterine contractions and genital bleeding were each considered by 28/32 centers (87.5%); regularity of contractions was considered by 23/32 (71.9%), and amount of bleeding by 16/32 (50.0%). Twenty‐six centers (81.3%) reported initiating intervention based on cervical length alone. Among the 22 centers that specified a cut‐off value, the values varied widely: ≤ 20 mm in 13 centers (40.6%), 21–25 mm in 8 (25.0%), and ≥ 26 mm in one (3.1%). Only three centers (9.4%) used an objective severity score, such as the PLI or TI. Cervical length cut‐off values were missing or not specified for 10 centers (Table 2).

**TABLE 2:** Diagnostic criteria and tocolytic drug use for threatened preterm labor.

Variable	Number of centers (%)
Diagnostic indicators^a^	
Cervical length measurement	32 (100)
Cervical dilation	32 (100)
Painful uterine contractions	28 (88)
Regularity of contractions	23 (72)
Genital bleeding	28 (88)
Amount of genital bleeding	16 (50)
Objective assessment score (e.g., PLI or TI)	3 (9)
Criteria for intervention^a^	
Short cervical length only	26 (81)
Cut‐off value for cervical length	
≤ 20 mm	13 (41)
21–25 mm	8 (25)
≥ 26 mm	1 (3)
Choice of tocolytic agents^a^	
First‐line drug	
Ritodrine hydrochloride	30 (94)
Magnesium sulfate	2 (6)
Second‐line drug	
Magnesium sulfate	27 (84)
Calcium channel blocker (CCB)	3 (9)
Ritodrine hydrochloride	2 (6)
Other practices	
Any use of CCB	15 (47)
Continuation of intravenous tocolysis after 34 weeks of gestation	26 (81)

*Note:* Cervical length cut‐off values were missing or not specified for 10 centers.

Abbreviations: CCB, calcium channel blocker; PLI, preterm labor index; TI, tocolysis index.

^a^ Multiple responses were allowed. Values are number of centers (%) (*n* = 32).

### 3.3 Association Between NICU Admission Policy and Tocolysis Duration

Centers with earlier gestational age thresholds for NICU admission (22–23 or 24–27 weeks) more frequently used short‐term tocolysis (short‐term only or short‐term plus long‐term), whereas all centers accepting NICU admissions only at ≥ 34 weeks used long‐term tocolysis (Figure 1). In multivariable logistic regression, a later NICU admission threshold was independently associated with use of long‐term tocolysis (adjusted OR [aOR], 1.38 per 1‐week increase; 95% CI, 1.04–1.83; *p* = 0.028), whereas number of formally registered NICU beds (aOR, 1.16; 95% CI, 0.90–1.50; *p* = 0.244) and region (Shikoku vs. Chugoku; aOR, 0.72; 95% CI, 0.12–4.52; *p* = 0.729) were not significant (Table 3). Sensitivity analyses showed the same direction when mixed‐policy centers were grouped with long‐term‐only centers (aOR, 1.50; 95% CI, 1.00–2.23; *p* = 0.048) or excluded (aOR, 1.53; 95% CI, 1.02–2.31; *p* = 0.042).

**FIGURE 1:**
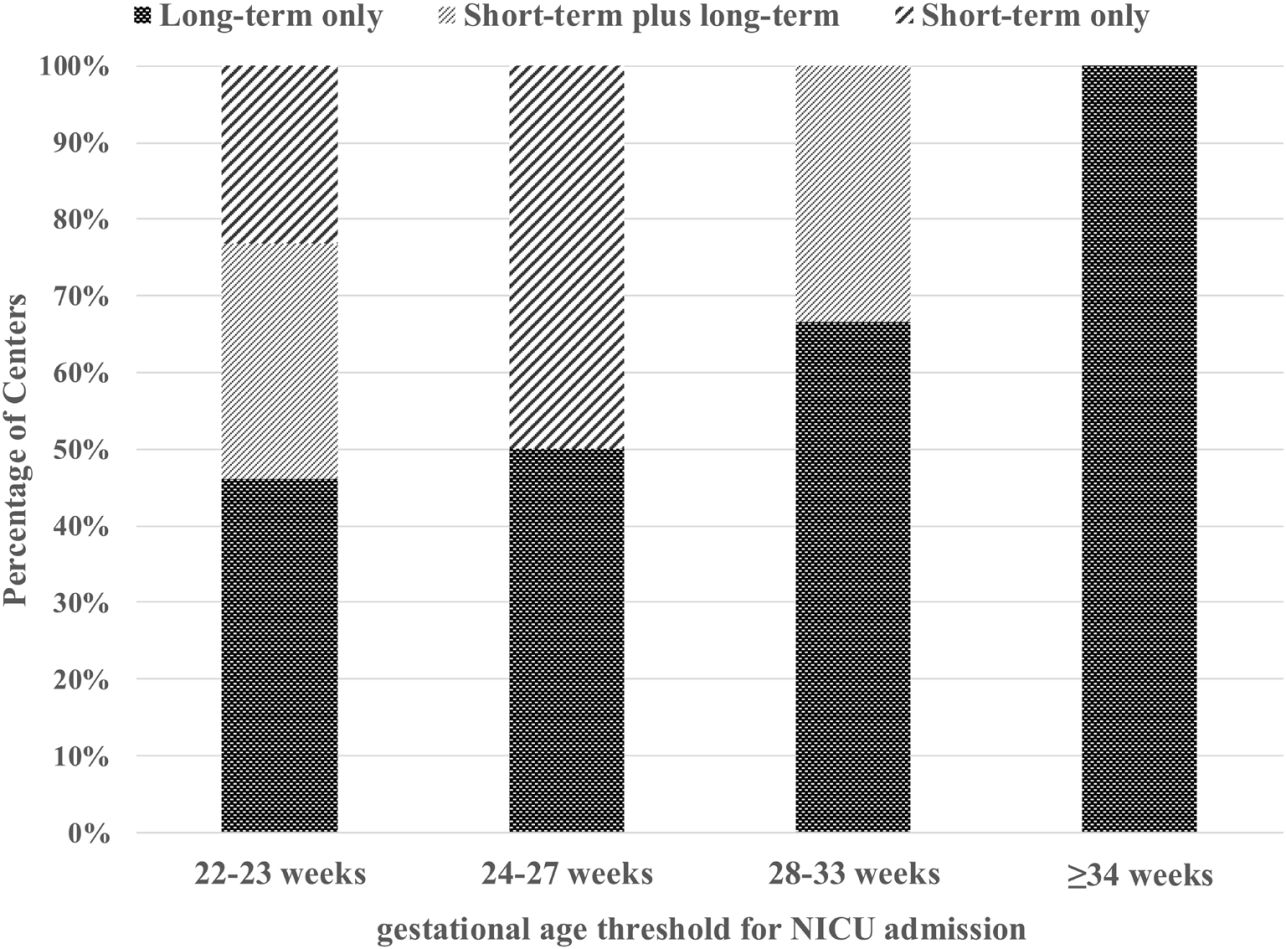
Gestational age threshold for NICU admission and routine intravenous tocolysis strategy for threatened preterm labor. Stacked bars show the percentage of centers in each NICU admission gestational age category (22–23, 24–27, 28–33, and ≥ 34 weeks). The short‐term policy group is partitioned into centers using short‐term only and short‐term plus long‐term. Centers using long‐term tocolysis only are shown separately. NICU, neonatal intensive care unit.

**TABLE 3:** Factors associated with use of long‐term tocolysis (facility‐level multivariable logistic regression).

Predictor	Adjusted odds ratio (aOR)	95% CI	*p*
NICU admission starting week (per 1‐week increase)	1.38	1.04–1.83	**0.028**
Formally registered NICU beds (per 1‐bed increase)	1.16	0.90–1.50	0.244
Region (Shikoku vs. Chugoku)	0.72	0.12–4.52	0.729

*Note:* Bold values indicate statistical significance (*p* < 0.05). Outcome: long‐term only versus short‐term (including short‐term only, short‐term plus long‐term); *n* = 32.

Abbreviations: aOR, adjusted odds ratio; CI, confidence interval; NICU, neonatal intensive care unit.

### 3.4 Drug Choice and Preterm Birth Rates

For first‐line tocolysis, 30/32 centers (93.8%) used ritodrine hydrochloride and 2/32 (6.3%) used magnesium sulfate. Calcium channel blockers were used by 15/32 centers (46.9%), mainly as second‐ or third‐line agents. The median facility‐level preterm birth rate < 34 weeks was 2.49%. This rate was higher in centers using short‐term tocolysis than in those using long‐term tocolysis (7.30% vs. 2.07%, *p* = 0.026). However, facility‐level preterm birth rates should be interpreted with caution because centers accepting extremely preterm infants used short‐term or mixed tocolysis policies more often.

## 4 Discussion

This cross‐sectional survey showed marked interfacility variation in diagnosis and management of TPL in western Japan (Chugoku–Shikoku region). The main finding was that the gestational age threshold for NICU admission was independently associated with the tocolysis strategy: lower and higher thresholds were associated with short‐term and long‐term use, respectively. These findings suggest that management strategies may be influenced by local neonatal care capacity, as well as by clinical evidence.

One possible explanation for the interfacility variation is the lack of a standardized diagnostic approach. More than 80% of centers reported initiating intervention primarily based on cervical length, but with varied cut‐off values across facilities. Spontaneous preterm birth can be predicted by cervical length, but the predictive performance is imperfect and only a minority of symptomatic women deliver shortly after presentation [8, 9, 10]. Thus, this prediction may lead to over‐triage, hospitalization, and intervention [2, 11]. Objective scoring systems such as the PLI or TI were used in only 9.4% of centers [12]. Symptoms such as uterine contractions and genital bleeding were considered at many centers, but were not incorporated into standardized frameworks. These findings suggest inconsistent diagnosis of TPL across centers, which may contribute to variation in management.

Management after diagnosis also differed from international recommendations. A long‐term‐only intravenous tocolysis policy was reported by 65.6% (21/32) of centers, in contrast to WHO and ACOG guidance that generally limits tocolysis to short‐term use [2, 3]. There is no evidence of a clear benefit of long‐term tocolysis for preventing preterm birth, but maternal adverse events remain a concern [2, 3]. Thus, use of β‐agonists such as ritodrine is restricted in Europe, where nifedipine and the oxytocin‐receptor antagonist atosiban are generally preferred [13, 14, 15]. The limited uptake of alternative agents and short‐term protocols in Japan may partly reflect regulatory and reimbursement conditions, differences in approved dosing regimens, and long‐standing clinical familiarity with ritodrine, although these mechanisms were not examined in this survey. Recent nationwide database studies suggest that prolonged ritodrine use remains common in Japan, although the practice may be gradually declining [4]. Our findings suggest that long‐term tocolysis in Japan may reflect both clinical custom and institutional and regional system constraints.

The association between the NICU admission threshold and the tocolysis strategy provides insights into underlying institutional factors. Centers capable of accepting very preterm infants can more readily follow a short‐term strategy consistent with international recommendations. In contrast, centers with higher gestational age thresholds for NICU admission may face pressure to prolong pregnancy until local neonatal care becomes feasible. In support of this interpretation, facilities in the short‐term group had more full‐time obstetricians and neonatologists, whereas those in the long‐term group more frequently required maternal transfer to higher‐level centers. Among the 20 centers that performed antepartum transfer to lower‐level centers, 14 reported a policy of continuing intravenous tocolysis for > 48 h as a bridge to transfer, either routinely or on a case‐by‐case basis. These findings suggest that long‐term tocolysis may represent an adaptive response to institutional capacity limitations, although this cross‐sectional association does not establish causality.

Geographic distance alone did not fully explain this pattern, since transfer distance did not differ significantly between facilities requiring maternal transfer in the two groups. Thus, long‐term tocolysis may be driven more by structural limitations of the referring facility than by distance itself. When neonatal acceptance criteria are restricted and resources are limited, long‐term tocolysis may function as a pragmatic strategy to avoid delivery before transfer or before a locally manageable gestational age is reached [16]. Therefore, in the context of the perinatal care system in Japan, prolonged tocolysis may be a compensatory response to the practical demands of in utero transfer and regional care coordination [17].

Several limitations of the study should be noted. First, the survey relied on self‐reporting, and institutional policies may not fully reflect actual practice. Second, we classified facilities by policy category rather than actual treatment duration in individual patients. Third, maternal and neonatal outcomes and patient‐level data were not collected, limiting direct assessment of safety, effectiveness, and differences in case mix. Fourth, diagnostic criteria were not standardized across centers. Fifth, detailed data on transfer timing and indications, transport availability, and receiving‐center decisions were unavailable and may have confounded the observed associations. Finally, generalizability of the findings beyond the Chugoku–Shikoku region is uncertain. Within these limitations, the NICU admission threshold was identified as an important structural factor related to prolonged tocolysis, showing the need for alignment between obstetric management and neonatal care capacity.

In conclusion, TPL management varied markedly among centers, and the gestational age threshold for NICU admission was associated with the tocolysis duration policy. These findings support nationwide surveillance of actual treatment duration and adverse events, prospective comparisons of short‐ and long‐term strategies, and clearer regional transfer protocols while considering regional perinatal system constraints.

## Author Contributions

**Naofumi Yamane:** writing – review and editing, writing – original draft, visualization, resources, investigation, data curation, formal analysis, methodology, project administration. **Yuko Teraoka:** conceptualization, writing – review and editing, writing – original draft, investigation, supervision. **Yurika Mukai:** conceptualization, supervision, writing – review and editing. **Kousuke Nakamoto:** writing – review and editing. **Yusuke Matoba:** writing – review and editing. **Yuriko Oomori:** writing – review and editing. **Katsuyuki Tomono:** writing – review and editing. **Tomomi Yamazaki:** writing – review and editing. **Kouji Banno:** writing – review and editing, supervision. **Ken Yamaguchi:** writing – review and editing, supervision.

## Funding

The article processing charge for this publication was supported by JSPS KAKENHI Grant Number 25K20091. No external funding was received for the conduct of this study.

## Ethics Statement

This study was approved by the Ethical Committee for Epidemiology of Hiroshima University (approval no. E2025‐0194).

## Consent

Completion of the questionnaire was taken to indicate consent to participate. Written patient consent was not required because no individual‐level or patient‐identifiable data were collected.

## Conflicts of Interest

The authors declare no conflicts of interest.

## Supporting information


**Figure S1:** Geographic distribution of perinatal centers in the Chugoku–Shikoku region of western Japan. Map pin‐shaped markers and triangles indicate comprehensive and regional perinatal centers, respectively. Solid markers represent centers from which responses were received (*n* = 32). Open markers represent centers from which responses were not received (*n* = 11; total 43 centers). Numbers on solid markers correspond to facilities listed in Table S1. Dashed lines indicate prefectural borders, and the inset map shows the study region in Japan (highlighted in black).

**Table S1:** List of participating perinatal medical centers (alphabetical order).
**Table S2:** Distribution of center designation by NICU admission gestational‐age threshold.
**Table S3:** Facility workforce and maternal transfer compared by routine intravenous tocolysis duration policy.

## Data Availability

The underlying data consist of facility‐level responses from 32 perinatal centers. The dataset includes combinations of institutional characteristics and care policies—including center designation, gestational‐age thresholds for neonatal intensive care unit admission, formally registered neonatal intensive care unit and growing care unit bed numbers, transfer practices, and tocolysis policy—that could permit re‐identification of participating centers even after direct identifiers are removed. During the publication and follow‐up confirmation process, participating centers were informed that facility specific detailed results would not be disclosed. Unrestricted public release of the facility‐level responses was not included in the approved study plan (Ethical Committee for Epidemiology of Hiroshima University; approval no. E2025‐0194). Accordingly, the underlying facility‐level dataset is not publicly available. Any external access to de‐identified data would require a separate review by the corresponding author and the Ethical Committee for Epidemiology of Hiroshima University and, where applicable, the participating institutions, together with an appropriate data‐use agreement. Aggregated data supporting the findings are provided in the article and its Supporting Information S1.
