# Supplementary material for: Interfacility Variation in Diagnosis and Tocolysis for Threatened Preterm Labor and Its Association With Gestational Age Threshold for Neonatal Intensive Care Unit Admission: A Cross‐Sectional Survey of Perinatal Centers in the Chugoku–Shikoku Region of Western Japan
Source: J Obstet Gynaecol Res. 2026 Oct 28;52(11):e70547. doi: 10.1111/jog.70547 (PMC13716503; doi:10.1111/jog.70547)
Supplement: Supplementary file 1 [file JOG-52-0-s002.docx]

**Supporting Information**

**Supplementary Figure S1.** **Geographic distribution of perinatal centers in the Chugoku-Shikoku region of western Japan.** Map pin-shaped markers and triangles indicate comprehensive and regional perinatal centers, respectively. Solid markers represent centers from which responses were received (n = 32). Open markers represent centers from which responses were not received (n = 11; total 43 centers). Numbers on solid markers correspond to facilities listed in Supplementary Table S1. Dashed lines indicate prefectural borders, and the inset map shows the study region in Japan (highlighted in black).

**Supplementary Table S1. List of participating perinatal medical centers (alphabetical order).**

| **Number** | **Perinatal medical center** |
| --- | --- |
| 1 | Chugoku Rosai Hospital |
| 2 | Ehime Prefectural Central Hospital |
| 3 | Ehime University Hospital |
| 4 | Hiroshima City Hiroshima Citizens Hospital |
| 5 | Hiroshima Prefectural Hospital |
| 6 | Hiroshima University Hospital |
| 7 | Imabari Prefectural Hospital |
| 8 | JA Onomichi General Hospital |
| 9 | JCHO Tokuyama Central Hospital |
| 10 | Kagawa University Hospital |
| 11 | Kawasaki Medical School Hospital |
| 12 | Kochi Health Sciences Center |
| 13 | Masuda Red Cross Hospital |
| 14 | Miyoshi Central Hospital |
| 15 | National Hospital Organization Fukuyama Medical Center |
| 16 | National Hospital Organization Higashihiroshima Medical Center |
| 17 | National Hospital Organization Iwakuni Clinical Center |
| 18 | National Hospital Organization Kure Medical Center and Chugoku Cancer Center |
| 19 | National Hospital Organization Okayama Medical Center |
| 20 | National Hospital Organization Shikoku Medical Center for Children and Adults |
| 21 | Niihama Prefectural Hospital |
| 22 | Okayama Red Cross Hospital |
| 23 | Okayama University Hospital |
| 24 | Shimane University Hospital |
| 25 | Takamatsu Red Cross Hospital |
| 26 | Tokushima Red Cross Hospital |
| 27 | Tottori Prefectural Central Hospital |
| 28 | Tsuchiya General Hospital |
| 29 | Uwajima City Hospital |
| 30 | Yamaguchi Prefectural Grand Medical Center |
| 31 | Yamaguchi Red Cross Hospital |
| 32 | Yamaguchi University Hospital |

**Abbreviations:** JA, Japan Agricultural Cooperatives; JCHO, Japan Community Healthcare Organization.

**Supplementary Table S2. Distribution of center designation by NICU admission gestational-age threshold.**

| **NICU admission threshold** | **All centers,**  **n (%)** | **Comprehensive centers,**  **n (% within category)** | **Regional centers,**  **n (% within category)** |
| --- | --- | --- | --- |
| 22–23 weeks | 13 (40.6) | 9 (69.2) | 4 (30.8) |
| 24–27 weeks | 2 (6.3) | 1 (50.0) | 1 (50.0) |
| 28–33 weeks | 9 (28.1) | 0 (0) | 9 (100) |
| ≥34 weeks | 8 (25.0) | 0 (0) | 8 (100) |
| Total | 32 (100) | 10 (31.3) | 22 (68.8) |

**Abbreviations:** NICU, neonatal intensive care unit

**Supplementary Table S3. Facility workforce and maternal transfer compared by routine intravenous tocolysis duration policy**

| **Characteristic** | **Total**  **(n = 32)** | **Short-term tocolysis**  **(n = 11)** | **Long-term tocolysis**  **(n = 21)** | **p value** |
| --- | --- | --- | --- | --- |
| Full-time obstetricians and gynecologists, median (IQR) | 7 (5–9) | 11 (9–14) | 7 (4–7) | **0.002**‡ |
| Board-certified obstetricians and gynecologists, median (IQR) | 6 (4–7) | 7 (7–12) | 5 (3–6) | **0.002**‡ |
| Board-certified maternal-fetal medicine specialists, median (IQR) | 2 (1–3) | 3 (2–4) | 2 (1–3) | 0.175‡ |
| Full-time pediatricians, median (IQR) | 7 (5–12) | 12 (7–16) | 6 (5–7) | 0.061‡ |
| Full-time neonatologists, median (IQR) | 3 (0–6) | 4 (3–6) | 1 (0–5) | **0.021**‡ |
| Requirement for maternal transfer to a higher-level center, n (%) | 21 (65.6) | 5 (45.5) | 16 (76.2) | 0.123† |
| Transfer distance for facilities requiring transfer (km)*, median (IQR) | 30.8  (11.2–59.4) | 11.7  (5.3–106.0) | 33.9  (16.5–55.2) | 0.905‡ |

**Abbreviations:** IQR, interquartile range; MFM, maternal-fetal medicine.

* Analyzed for the 21 facilities that require maternal transfer: Short-term tocolysis group (n = 5); Long-term tocolysis group (n = 16).

† Fisher exact test.

‡ Mann-Whitney U test.

Bold values indicate statistical significance (p < 0.05).
